# A decline in transcript abundance for *Heterodera glycines *homologs of *Caenorhabditis elegans uncoordinated *genes accompanies its sedentary parasitic phase

**DOI:** 10.1186/1471-213X-7-35

**Published:** 2007-04-19

**Authors:** Vincent P Klink, Veronica E Martins, Nadim W Alkharouf, Christopher C Overall, Margaret H MacDonald, Benjamin F Matthews

**Affiliations:** 1 United States Department of Agriculture, Soybean Genomics and Improvement Laboratory, Beltsville, MD 20705, USA; 2 Graduate School of Biotechnology Studies, University of Maryland University College, Adelphi, MD 20783, USA; 3 Jess and Mildred Fisher College of Science and Mathematics, Department of Computer and Information Sciences, Towson University, 7800 York Road, Towson, Maryland 21252, USA; 4 Department of Bioinformatics and Computational Biology, George Mason University, Manassas 20110, VA, USA

## Abstract

**Background:**

*Heterodera glycines *(soybean cyst nematode [SCN]), the major pathogen of *Glycine max *(soybean), undergoes muscle degradation (sarcopenia) as it becomes sedentary inside the root. Many genes encoding muscular and neuromuscular components belong to the *uncoordinated *(*unc*) family of genes originally identified in *Caenorhabditis elegans*. Previously, we reported a substantial decrease in transcript abundance for *Hg-unc-87*, the *H. glycines *homolog of *unc-87 *(calponin) during the adult sedentary phase of SCN. These observations implied that changes in the expression of specific muscle genes occurred during sarcopenia.

**Results:**

We developed a bioinformatics database that compares expressed sequence tag (est) and genomic data of *C. elegans *and *H. glycines *(CeHg database). We identify *H. glycines *homologs of *C. elegans unc *genes whose protein products are involved in muscle composition and regulation. RT-PCR reveals the transcript abundance of *H. glycines unc *homologs at mobile and sedentary stages of its lifecycle. A prominent reduction in transcript abundance occurs in samples from sedentary nematodes for homologs of actin, *unc-60B *(cofilin), *unc-89*, *unc-15 *(paromyosin), *unc-27 *(troponin I), *unc-54 *(myosin), and the potassium channel *unc-110 *(*twk-18*). Less reduction is observed for the focal adhesion complex gene *Hg-unc-97*.

**Conclusion:**

The CeHg bioinformatics database is shown to be useful in identifying homologs of genes whose protein products perform roles in specific aspects of *H. glycines *muscle biology. Our bioinformatics comparison of *C. elegans *and *H. glycines *genomic data and our *Hg*-*unc-87 *expression experiments demonstrate that the transcript abundance of specific *H. glycines *homologs of muscle gene decreases as the nematode becomes sedentary inside the root during its parasitic feeding stages.

## Background

Many aspects of muscle development and maintenance were elucidated through genetic screens in the free-living nematode *C. elegans *[1-3]. Subsequently, homologs of these genes can be found in other organisms using bioinformatics, allowing a broader understanding of how they may function. Most of the studies investigating muscle development and maintenance in *C. elegans *focus on the location of the proteins or examine their genetic and biochemical nature. There is less work on determining what happens to these muscle proteins (and hence changes in muscle composition) over the course of normal development [4,5].

The formation, maintenance and degradation (wasting) of muscles involve a suite of proteins, many that are highly conserved [6-12]. The wasting of muscles over time is known as sarcopenia [13]. Sarcopenia is attributed to many factors including aging, hormone balance, decreased physical activity, malnutrition and oxidative stress [14,15]. In *C. elegans*, contraction-related injury of pharynx muscles causes sarcopenia [15]. Sarcopenia normally occurs slowly over the lifetime of an organism. However, several genetic diseases such as Duchenne muscular dystrophy (DMD) generate similar, but hastened, wasting phenotypes [16]. In these cases, however, muscles can never regenerate due to their genetic predisposition. While genetic disorders may mimic sarcopenia, some organisms undergo rapid muscle wasting that is normal to specific stages of their lifecycle. Some reports indicate that this targeted degradation of muscle proteins is actually adaptive and not pathological. Thus, sarcopenia provides resources that can be utilized for other metabolic functions. [17]. The decrease in muscle protein content, presumably, would be accompanied by a decrease in transcription of those genes.

Our lab has focused on the interaction between the parasitic nematode *Heterodera glycines *and *Glycine max *[18-26]. *H. glycines *is the major parasite of *G. max *and is responsible for causing losses approaching a billion dollars annually for the agricultural industry in the U.S. [27]. Thus, knowledge on the regulation of muscle development is not only relevant to muscle senescence, probable nutrient recycling, for better understanding its developmental biology and for understanding parasitism, but may, in turn, lead to better nematode control measures. The *C. elegans-H. glycines *database (CeHg database) allows us to assign function and better understand *H. glycines *genes [26]. The CeHg database connects the vast information on *C. elegans *gene function with *H. glycines *expressed sequence tags (ests) to rapidly identify essential *H. glycines *genes that could be attributed to a specific defect (i.e. lethality [26]). In fact, one *H. glycines *gene predicted to be essential using this bioinformatics approach, was shown to be essential through gene silencing using RNAi [26]. RNAi decreased the transcript abundance of the targeted gene, causing nematode death. [26]. We believe that the CeHg database can identify genes important to muscle biology and sarcopenia in *H. glycines *during its lifecycle.

The genetically-defined *uncoordinated *(*unc*) genes perform many functions in *C. elegans*. The protein products of the *unc *genes are involved in muscle focal adhesion, architecture and stimulation (via neuromuscular connections). However, null alleles of *unc *genes can exhibit Paralyzed Arrested at Two-fold stage (*pat*) phenotypes. The *unc *mutants all display uncoordinated motion, slow movement, or paralysis [3]. The *unc *family of mutants contains 114 different members [3,28]. We believe that much of the muscle degeneration observed in *H. glycines *would likely involve transcriptional regulation of *H. glycines *homologs of *unc *genes whose protein products are involved in (1) the acto-myosin complex, (2) muscle focal adhesion or (3) other aspects of muscle composition and regulation.

In this paper, we use an in-house bioinformatics database [26] to identify *H. glycines *homologs of *unc *genes. We identify *H. glycines *homologs of genes composing (1) acto-myosin complex, (2) muscle focal adhesion and (3) other aspects of muscle composition and regulation. We determine the transcript abundance of these *H. glycines unc *homologs using RT-PCR. Gene expression for many of these *Hg*-*unc *homologs is high during the mobile phase of *H. glycines *development and is lower during the sedentary phase of *H. glycines *life cycle.

## Results

### Identification of unc genes in *H. glycines*

**Figure 1:**
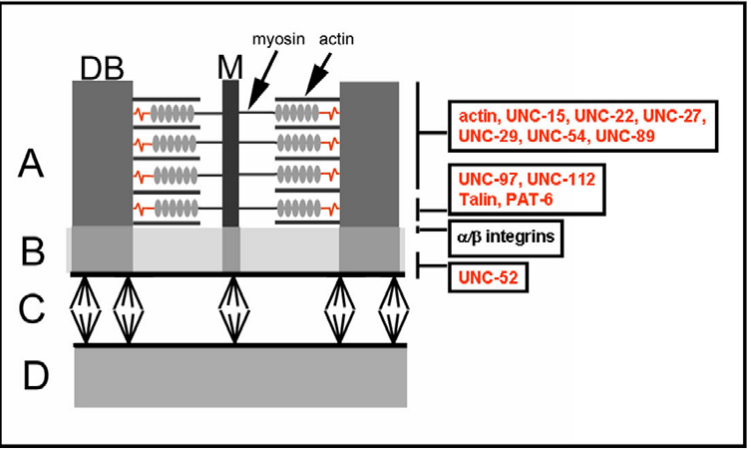
A Diagrammatic representation of a muscle cross-section shows the dense body (DB) and M-line (M). This section is divided into four regions; A, muscle; B, basal lamina; C, hypodermis; D, cuticle. The M-line is composed of *unc-89*. Arrows point toward the position of actin and myosin. The *unc *genes studied and their relative positions are provided to the right. Genes in red are part of this study. Genes in black were not studied. Figure adapted from [6, 73, 74].

**Figure 2:**
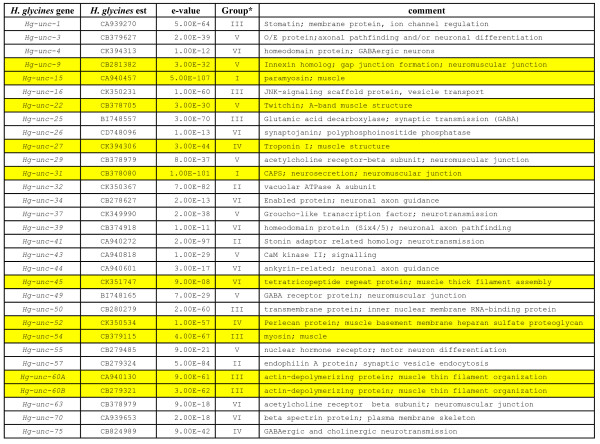
*H. glycines *est homologs of *C. elegans unc *genes. Column headings provide the *unc *gene, *H. glycines *est sequence, e-value, and gene function. The genes are divided into six groups (Group I-VI) based on the following arbitrarily selected significance intervals: E-values between 0 and 1E-100 (Group I), between 1E-100 and 1E-80 (Group II), between 1E-80 and 1E-60 (Group III), between 1E-60 and 1E-40 (Group IV), between 1E-40 and 1E-20 (Group V) and E-values > 1E-20 (Group VI) [26]. In yellow are the genes used for RT-PCR experiments.

**Figure 3:**
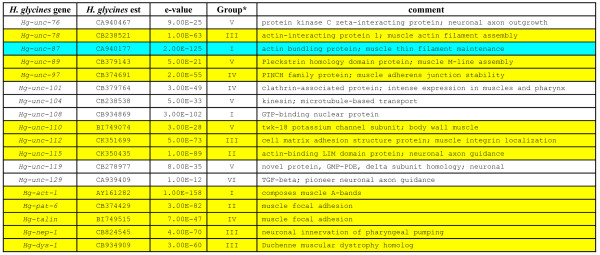
*H. glycines *est homologs of *C. elegans unc *genes. Column headings provide the *unc *gene, *H. glycines *est sequence, e-value, and gene function. The genes are divided into six groups (Group I-VI) based on the following arbitrarily selected significance intervals: E-values between 0 and 1E-100 (Group I), between 1E-100 and 1E-80 (Group II), between 1E-80 and 1E-60 (Group III), between 1E-60 and 1E-40 (Group IV), between 1E-40 and 1E-20 (Group V) and E-values > 1E-20 (Group VI) [26]. In yellow are the genes used for RT-PCR experiments. The previously published *H. glycines *muscle gene, *Hg-unc-*87 [19] is presented in cyan.

*Unc *gene products compose various parts of the body wall muscle (Fig. 1). We identified 45 *H. glycines *est homologs of *C. elegans unc *genes (*Hg-unc*) (Figs. 2 and 3). We confirmed the identification of the *Hg-unc *genes by performing manual blast searches of the *C. elegans unc *genes in Genbank. We also identified other *H. glycines *ests (dystrophin [*Hg-dys-1*], neprilysin [*Hg-nep-1*], actin [*Hg-act-1*], talin [*Hg-talin*], *pat-6 *[*Hg-pat-6*]) whose mutants exhibit *unc *phenotypes or whose protein products interact with UNC proteins in *C. elegans*. However, the original *unc *mutant screens did not identify them (Figs. 2 and 3).

### Transcript abundance of *unc *genes involved in thin filament composition and maintenance

**Figure 4:**
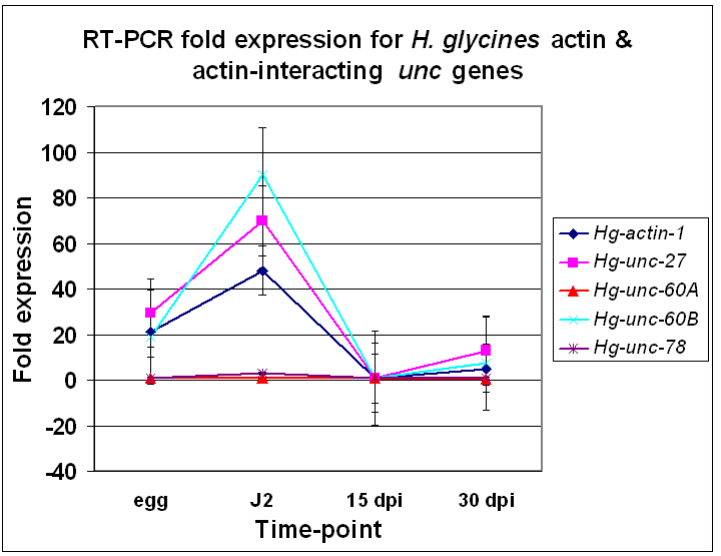
RT-PCR fold expression for *H. glycines *actin and actin-interacting *unc *genes. RT-PCR of *Hg-act-1*, *Hg-unc-27*, *Hg-unc-60A/B*, and *Hg-unc-78 *ESTs homologous to *C. elegans unc *genes showing the fold expression (y-axis) plotted against the time-point (egg, J2, 15 dpi and 30 dpi).

We identified a decline in transcript abundance for *Hg-unc-87 *during the transition from the mobile to the sedentary phase of the *H. glycines *lifecycle [19]. This observation indicates that microfilament degradation occurs during muscle wasting. Bioinformatics analyses identified several *H. glycines *ests that are homologous to *C. elegans *thin filament genes, including *Hg-act-1*, *Hg-unc-27*, *Hg-unc-60A*, *Hg-unc-60B *and *Hg-unc-78 *(Figs. 2 and 3). RT-PCR revealed a substantial decline in actin transcript abundance occurring between the J2 stage and 15 dpi nematodes (Fig. 4). RT-PCR revealed a substantial decline in transcript abundance of *Hg-unc-27 *occurring between the J2 stage and 15 dpi nematodes (Fig. 4). We examined the expression profile of the two *Hg*-*unc-60 *isoforms (A and B) and *Hg-unc-78*. *Hg-unc-60A *is the non-muscle *unc-60 *isoform, while *Hg-unc-60B *is the muscle-specific isoform) RT-PCR of *Hg-unc-60A *reveals little change in transcript abundance occurring throughout the *H. glycines *lifecycle (Fig. 4). However, RT-PCR reveals a substantial decline in transcript abundance occurring for *Hg-unc-60B *between the J2 stage and 15 dpi nematodes (Fig. 4). The decline in transcript abundance for *Hg-unc-60B *and not *Hg-unc-60A*, occurring between the J2 stage and 15 dpi nematodes, is in agreement with its muscle-specific activity. RT-PCR reveals little change in transcript abundance occurring throughout the *H. glycines *lifecycle for *Hg-unc-78 *(Fig. 4).

### Transcript abundance of *unc *genes involved in thick filament composition and maintenance

**Figure 5:**
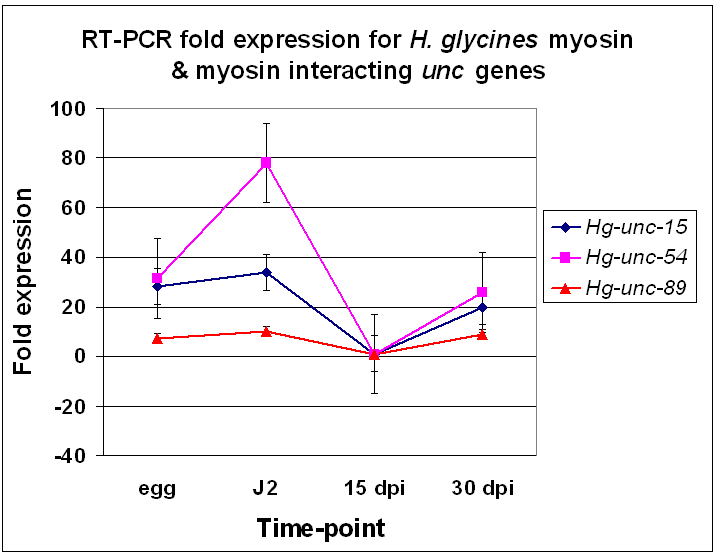
RT-PCR fold expression for *H. glycines *myosin and myosin interacting *unc *genes. RT-PCR of *Hg-unc-15 *(paramyosin), *Hg-unc-27*, *Hg-unc-54 *(myosin) and *Hg-unc-89 C. elegans unc *genes showing the fold expression (y-axis) plotted against the time-point (egg, J2, 15 dpi and 30 dpi).

Bioinformatics analyses identified *H. glycines *ests homologous to *C. elegans *thick filament genes (Figs. 2 and 3). Transcript abundance of *H. glycines unc *genes whose homologous gene products compose thick filaments in *C. elegans *was measured using RT-PCR. RT-PCR revealed a substantial decline in transcript abundance of *Hg-unc-15 *and *Hg-unc-54 *occurring between the J2 stage and 15 dpi nematodes (Fig. 5). Furthermore, transcript levels of *Hg-unc-89 *also decline between the J2 stage and 15 dpi nematodes (Fig. 5).

### Transcript abundance of focal adhesion complex genes

**Figure 6:**
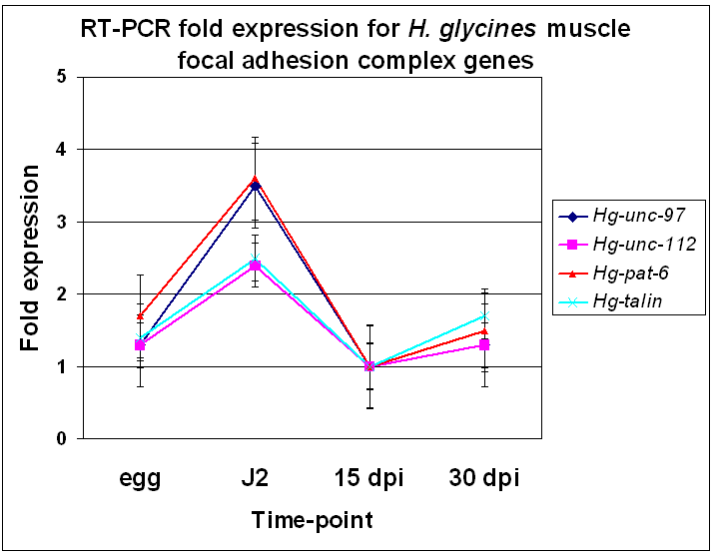
RT-PCR fold expression for *H. glycines *muscle focal adhesion complex genes. Results of transcript levels of *Hg-unc-97*, *Hg-unc-112*, *Hg-pat-6 *and *Hg-talin *showing the fold expression (y-axis) plotted against the time-point (egg, J2, 15 dpi and 30 dpi).

Bioinformatics analyses also identified *H. glycines *ests homologous to *C. elegans *focal adhesion genes (Figs. 2 and 3). *H*g-*unc-97 *transcript levels decrease in abundance between the J2 and 15 dpi nematodes, as shown by RT-PCR (Fig. 6). We explored the focal adhesion complex further by examining the transcript abundance of *Hg-unc-112*, *Hg-pat-6 *and *Hg-talin*. *Hg-unc-112*, *Hg-pat-6 *and *Hg-talin *transcript levels decrease between the J2 stage and 15 dpi nematodes (Fig. 6). Bioinformatics analyses did not identify homologs of other focal adhesion complex proteins.

### RT-PCR of *H. glycines *ests homologous to *C. elegans unc *genes

**Figure 7:**
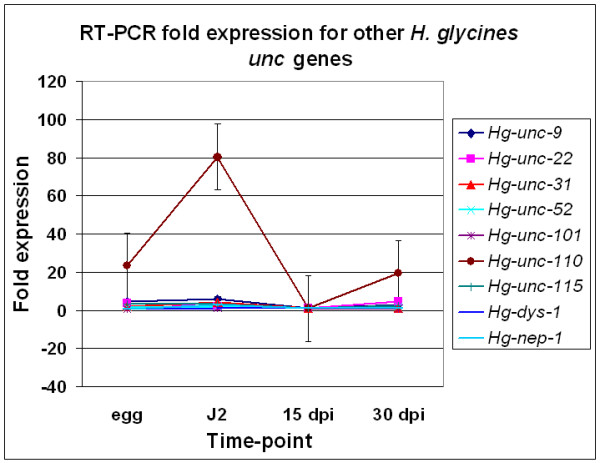
RT-PCR fold expression for the other *H. glycines unc *genes. RT-PCR of *Hg-unc-9, Hg-unc-22, Hg*-*unc-31*, *Hg*-*unc-52*, *Hg*-*unc-101*, *Hg*-*unc-115*, *Hg*-*unc-110, Hg-dys-1 *and *Hg-nep-1 *showing the fold expression (y-axis) plotted against the time-point (egg, J2, 15 dpi and 30 dpi).

Bioinformatics analyses identified *H. glycines *ests homologous to *C. elegans *genes whose protein products function in other aspects of muscle biology (Figs. 2 and 3) These *H. glycines unc *genes include *Hg-unc-9*, *Hg-unc-22 *(twitchin), *Hg-unc-31*(CAPS), *Hg-unc-52 *(perlecan), *Hg-unc-101*, *Hg-unc-115*, *Hg-unc-110 *(*Hg-twk-18*), *Hg-dys-1*, and *Hg-nep-1*. RT-PCR analysis indicated that modest changes in transcript abundance occur for *Hg-unc-9*, *Hg-unc-22, Hg-unc-31*, *Hg*-*unc-52*, *Hg-unc-101*, *Hg-unc-115, Hg-dys-1*, and *Hg-nep-1 *between the J2 stage and 15 dpi nematodes (Fig. 7). RT-PCR also indicated that *Hg-unc-110 *transcript abundance decreases substantially between the J2 stage and 15 dpi nematodes (Fig. 7).

## Discussion

### Use of the CeHg database to identify *unc *genes in *H. glycines*

Body wall muscle degradation accompanies the sedentary phase of *H. glycines *as it feeds from the syncytium. Thus, important transcriptional, translational, and post-translational changes occur at this time. We began our analysis of *H. glycines *muscle wasting by identifying *H. glycines *homologs of *C. elegans *muscle genes. We then identified the transcript abundance of those genes whose protein products compose the acto-myosin complex, muscle focal adhesion complex, neuromuscular connections and potassium channels.

The acto-myosin complex is composed of interdigitating thin and thick filaments that are bundled by UNC-87 [29-31]. Previously, we observed a decline in transcript abundance of *Hg-unc-87 *[19]. This demonstrated that depletion of components of the acto-myosin complex may occur during the sedentary phase of the *H. glycines *lifecycle. Actin and troponin I (*unc-27*) are primary components of the thin filaments. Actin is not classified as an *unc *gene. However, the *unc-92 *mutant of *C. elegans *maps to the actin locus and may actually be actin. Recently, Willis et al. [11], found that the actin family in *C. elegans *is composed of five highly conserved isoforms (act-1–5) and yields an *unc *phenotype [11]. Only one actin gene is present in *H. glycines *[32]. *Unc-27 *is involved in thin filament maintenance. UNC-27 forms a complex with troponin C (PAT-10) and troponin T [33-35] to accomplish calcium-dependent regulation of the acto-myosin interaction [36]. Mutant *unc-27 *disorganizes dense body positioning. Mutant *unc-27 *causes less well-defined sarcomeres with small regions of thin filaments interspersing within the overlap of A-bands [37]. We found that, as expected, *Hg-act-1 *and *Hg-unc-27 *experience a substantial decrease in expression between J2 and 15 dpi nematodes

The dynamic nature of actin filaments is under control of the actin interacting proteins UNC-60 and UNC-78. UNC-60 is the actin depolymerizing factor (ADF) cofilin. Mutations in *unc-60 *cause disorganization in muscles by preventing bundling of thin filaments with myosin into functional contractile units [38]. However, in *C. elegans *the *unc-60 *gene actually encodes two completely different protein products. UNC-60A and UNC-60B are products of SUP-12-dependent alternative splicing [39]. UNC-60A and UNC-60B perform distinct roles in actin dynamics [40]. UNC-60A is the non-muscle cofilin isoform while the UNC-60B is the muscle-specific cofilin. Like *C. elegans *[41], *H. glycines *has orthologous mRNA sequences for both *unc-60A *and *unc-60B*. UNC-78 is the muscle-specific actin interacting protein (AIP). UNC-78 works in concert with UNC-60B to depolymerize microfilaments into actin monomers [40-43]. Unlike *unc-60*, *unc-78 *does not appear to have multiple splice variants that perform distinct muscle and non-muscle functions. Our examination of *unc-60*, indicates that the muscle-specific *unc-60 *isoform, *Hg-unc-60B*, exhibits a substantial decrease in transcript abundance between J2 and 15 dpi nematodes. This is consistent with its important role in muscle organization. As expected, the non-muscle *unc-60 *isoform, *Hg-unc-60A*, does not exhibit changes in transcript abundance during the *H. glycines *lifecycle. *Hg-unc-78*, a gene whose protein product regulates actin polymerization does not experience a substantial change in gene expression during the transition from the J2 stage to the sedentary phase. These observations, taken together with the substantial decrease in transcript abundance of the actin bundling muscle gene *Hg-unc-87 *[19], indicate that major changes in transcript abundance occur for *Hg-act-1*, *Hg-unc-27 *and the protein products (i.e. *Hg*-UNC-60B) that regulate actin in the body wall muscles.

### Myosin metabolism and muscle mass

Thick filaments are major components of muscles. In *C. elegans*, myosin (UNC-54), paromyosin (UNC-15) and myosin heavy chain A (MYO3) compose thick filaments. Thick filaments are anchored to the M-line on one side and bound to the dense body on the side by the protein titin [44]. UNC-89 organizes muscles by assembling thick filaments into A-bands [45]. UNC-89 is also essential for M-line assembly [45]. There are three UNC-89 isoforms n *C. elegans *[45]. Our RT-PCR analysis demonstrates a decrease in transcript abundance for *Hg*-*unc-15*, *Hg*-*unc-54 *and *Hg*-*unc-89 *occurring during muscle wasting. Thus, a decrease in transcript abundance for actin and myosin gene products occurs during muscle wasting as nematodes are becoming sedentary during their parasitic feeding stages. Loss of muscle mass occurs in mutants for muscle genes. For example, loss of muscle mass is a characteristic of DMD, caused by *dys-1 *mutants. However, in *C. elegans*, DMD-like muscle defects also require dystrobrevin (DYB-1). A microarray experiment explored the complexities of the *dys-1 *mutant background [46]. Microarrays of *dys-1 *revealed 44 total probe sets are induced while 71, including *unc-89*, are suppressed [46]. It is not clear how a decrease in transcript abundance of *unc-89 *is involved in DMD. Differential expression of myosin transcripts was also observed in that study [46].

### Muscle focal adhesion complex degradation and muscle mass

The focal adhesion complex is composed of numerous proteins. In *C. elegans*, UNC-97 is part of the PINCH family of proteins that are composed of five **l**in-11 **i**sl-1 **m**ec-3 [47] (LIM) domains. LIM domains are found in proteins with wide-ranging cellular roles including fate determination of cells, cytoskeleton, organ development and intracellular trafficking. LIM domains have a consensus amino acid sequence CX_2_CX_16–23_HX_2_CX_2_CX_2_CX_16–23_CX_2–3_(C, H, D) and are putative structural motifs for binding zinc [47,48]. The tandem nature of the LIM domains provides potential for multiple protein-protein interactions. The LIM domain-containing protein family is characterized by its ability to attach to body wall muscles, vulval muscles, and mechanosensory neurons [49,50]. *C. elegans *UNC-97 does this by positioning itself with the β-integrin PAT-3 of muscles [50]. A splice-site mutation of *unc-97 *displays an *unc *phenotype, while the phenotype displayed by RNAi is *pat *and is embryonic lethal. Thus, UNC-97 is necessary for assembly and stability of muscular adherens junctions [50]. In *C. elegans*, the structural components that secure myofibers to the extracellular matrix, such as integrin, vinculin, talin, and α-actinin are conserved. This complex is similar in organization to adherens junctions in tissue culture cells [50]. The depletion of UNC-97 function leads to the disruption of these focal adhesion structures as well as of the mechanosensory neurons [50]. Further biochemical studies show the integrin-linked kinase (PAT-4) binds UNC-97. PAT-4 binds at the first Zn^+2^-binding module of the first LIM domain through an interaction with the N-terminal-most region of ankyrin repeat 1 (ANK1). In *C. elegans*, a biochemical interaction occurs between the sex-linked UNC-98 and UNC-97 [51]. This interaction requires the first two LIM domains of UNC-97 and all four Zn^+2^-fingers of UNC-98 [51]. The biological role for LIM domain 4, the most highly conserved LIM domain of UNC-97, remains elusive. Other proteins composing focal adhesion complex are UNC-112, a novel protein required for integrin localization [52]; PAT-6, responsible for assembling integrin adhesion complexes [53] and TALIN, a protein requiring β-integrin for its incorporation into focal adhesion-like structures [54].

Our bioinformatics analysis identified the focal adhesion complex genes *Hg-unc-97*, *Hg-unc-112*, *Hg-talin*, and *Hg-pat-6*. A modest decrease in transcript abundance occurs for these genes between the J2 and 15 dpi nematodes. These results demonstrate that the deterioration of focal adhesion sites, by depletion of *Hg-*UNC-97, *Hg-*UNC-112, *Hg-*TALIN, and *Hg-*PAT-6, may not be a major contributor to body wall muscle wasting.

### *Unc *metabolism and muscle mass

*Unc *gene products perform other important roles in muscle biology. For example, UNC-9, is a neuromuscular gap junction protein [55]; UNC-22 (twitchin) is involved in muscle A-band structure [56,57]; UNC-31(CAPS) is involved in the neuromuscular junction and neurosecretion [58], UNC-52 (perlecan), is a muscle basement membrane heparan sulfate proteoglycan protein [59]. Mutant *unc-52 *can also exhibit a *pat *phenotype, depending on the mutant allele [60]; UNC-101, is a clathrin-associated protein having intense expression in muscles and pharynx; UNC-115, is an actin-binding, LIM domain containing protein that is involved in neuronal axon guidance [61,62] and UNC-110, is a potassium channel subunit protein involved in body wall muscle control [63]. Dystrophin (*dys-1*) and neprilysin (*nep-1*) are other genes whose mutants exhibit *unc*-like phenotypes. The *dys-1 *gene is the *C. elegans *Duchenne muscular dystrophy homolog. The *dys-1 *gene product is part of the dystrophin-glycoprotein complex that is found in the plasma membranes of muscle cells. The dystrophin-glycoprotein complex is responsible for linking the intracellular cytoskeleton to the extracellular matrix and thought to be important for organizing signal molecules [64] and mechanical integrity [65]. The *nep-1 *gene product is involved in the neuronal network of pharyngeal pumping [66].

We observed only modest changes in gene expression occurring for many of the other *H. glycines unc *gene homologs. However, the *H. glycines *homolog of the body wall muscle-specific potassium channel protein, UNC-110, experiences a substantial decrease in transcript abundance between the J2 and 15 dpi nematodes. This decrease in transcript abundance is similar to the decrease in transcript abundance shown for the acto-myosin genes. It is not clear how a decrease in abundance for potassium channel proteins like *Hg-*UNC-110 would contribute to the sedentary nature of *H. glycines *during later stages of parasitism. However, potassium channels do perform major roles in muscle function in *C. elegans *[63,67,68]. At least 42 genes exist in the *C. elegans *genome that encode two-P domain K(+) (TWK) channels. These K+ channel subunits contain four transmembrane domains and two pore regions. *Unc-110 *is *twk-18 *and in *C. elegans*, TWK-18 localizes to the body wall muscle. The *twk-18 *mutant confers both uncoordinated movement and paralysis, probably a consequence of their expression of much larger potassium currents [63]. The locomotion defect caused by mutants in K+ channel genes [63,67,68] indicates how the substantial decrease in *Hg-unc-110 *transcript abundance could contribute to the lack of mobility in *H. glycines *during its sedentary phase.

## Conclusion

Our results demonstrate a decrease in transcript abundance for a specific subset of *H. glycines *homologs of *unc *genes. This decrease in transcript abundance correlates to the sedentary phase of the *H. glycines *lifecycle. We show a substantial decrease in transcript abundance of genes composing the acto-myosin complex and also for the K+ channel homolog *Hg-unc-110 *during muscle wasting. Deterioration of focal adhesion sites does not appear to account for much of the muscle mass lost during sarcopenia in *H. glycines*.

## Methods

### Plant and nematode materials and RNA isolation

Plant and nematode materials were grown at the United States Department of Agriculture Soybean Genomics and Improvement Laboratory as described previously [21] according to the moisture replacement system [69]. Our study uses eggs and J2 stage nematodes that are composed of male and female nematodes. This was done because at this time it is not possible to distinguish between immature male and females at the egg and J2 stages. Hatching and subsequent migration of J2s are identical between male and female nematodes. Thus, it is likely that, concerning muscle biology, males and females are nearly identical at the egg and J2 stages. The later stages we use (15 and 30 dpi) are composed entirely of sedentary parasitic female nematodes because that was the focus of this study.

Briefly, *G. max *cv. Peking seeds were grown in a sand mix in standard greenhouse conditions. To promote hatching, eggs from the *H. glycines *isolate TN8 (susceptible reaction) were incubated in sterile water at room temperature on a rotary shaker at 25 rpm. After two days on the rotary shaker, the J2s were collected and concentrated by centrifugation to approximately 5,200 J2/ml. Replicate experiments were performed and completed by running one experiment to completion and then collecting data. A second experiment was then run at approximately one month later after the first experiment was completed. Thus, we isolated different isolations of *H. glycines *eggs, J2, 15 dpi and 30 dpi nematodes for each experiment. Four plants contained in a beaker were inoculated with 5,200 J2 nematodes. For RT-PCR experiments, recovery of the 15 and 30 dpi *H. glycines *samples from *G. max *roots was performed according to [69] and also done previously in our lab [19]. Briefly, *H. glycines*-infected *G. max *roots grown for either 15 or 30 dpi were dipped into water to remove sand. At 15 and 30 dpi, female *H. glycines *are partially emerged from the root, facilitating collection of pure nematode samples. Roots were lightly massaged to liberate female *H. glycines *into a sieve of 150 μm pore size (VWR Scientific; Bridgeport, NJ). This filtration step would remove any additional male nematodes that were remaining in the root. To obtain egg and J2 samples, mature females were harvested at 30 dpi and crushed. The eggs were sifted through a 250 μm sieve and captured onto a 25 μm sieve. The eggs were hatched for two days in distilled water on a rotary shaker at 25 rpm. Pure J2 suspensions are made by sifting them through a 41 μm nylon mesh and collected with a low-speed centrifugation. The RT-PCR samples (J2, 15 dpi female, 30 dpi female or eggs) were flash-frozen in liquid nitrogen and ground to a fine powder using mortar and pestle chilled in liquid nitrogen. Total RNA extraction was performed using the method of Mujer et al. [70].

### CeHg database analysis

The CeHg bioinformatics database [26] is a database containing 300,773 ests and 6,630 genomic sequences from *C. elegans *and 24,438 ests and 231 genomic sequences from *H. glycines *(May, 2006). These *C. elegans *and *H. glycines *sequences were used to create a local database using SQLServer2000. The sequences were imported into our local database. Subsequently we created a unigene set using the contig assembly program Seqman (DNAStar Inc.; Madison, WI) resulting in 3,782 contigs of 2 or more sequences and 4,522 singletons for *H. glycines*. These sequences were then blasted against the local *C. elegans *database. Parsing of the results of the blast searches was done with customized Perl scripts. These scripts extracted the best hits from the blast results, E-value, score and identities values. The parsed results were imported back into the database. SQL scripts were written to query the CeHg database for *C. elegans *genes having high homology. Our data base was then linked to WormBase [71] and PubMed [72] to identify *H. glycines *ests homologous to *C. elegans unc *genes. These results then were confirmed by performing manual blast searches of each *C. elegans unc *gene against the *H. glycines *sequences.

### RT-PCR

**Figure 8:**
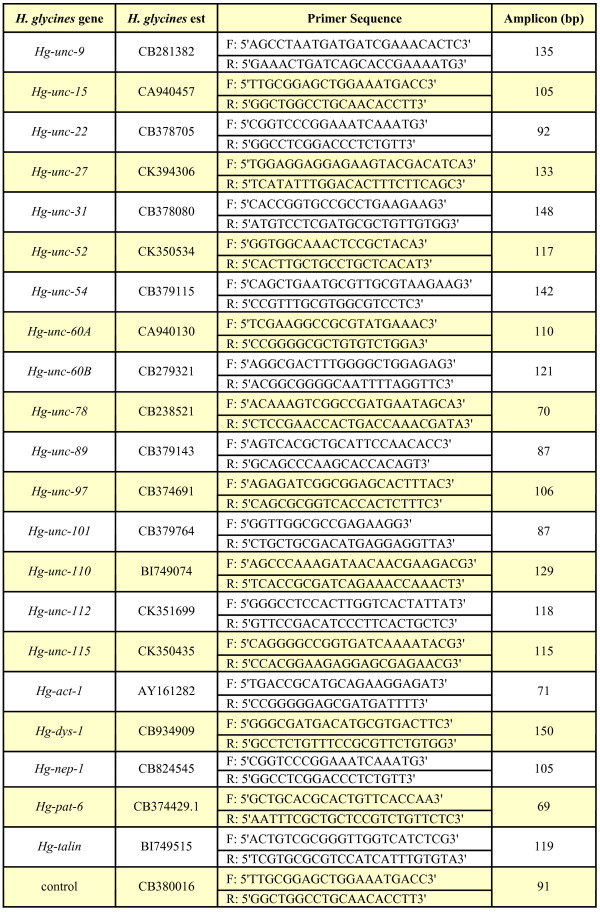
PCR primer pairs for RT-PCR expression analyses. For the RT-PCR primers, the Genbank match for each *unc *homolog is provided. The amplicon length is provided in base pairs.

RNA was extracted from nematodes as previously described and treated with DNase I to remove genomic DNA. The cDNA was reversed transcribed from RNA using SuperScript First Strand Synthesis System for RT-PCR (Invitrogen; Grand Island, NY) with oligo d(T) as the primer according to manufacturer's instructions. All the primer sets were initially tested for specificity with a mixture of RNAs for all stages of nematodes. Genomic DNA contamination was assessed by PCR as described previously [19]. We performed this experiment to identify any contaminating genomic DNA that may exist in our cDNA. We used *Hg-unc-87 *PCR primers (forward primer: 5'GACAACACGGAGATTCCACTTCAG3'; reverse primer, 5'CTGGTCTGGTCGATGCTCTGCTC3') that amplify different size fragments in the presence of genomic DNA as compared to pure cDNA. RT-PCR reactions containing no template and reactions using RNA processed in parallel but with no Superscript reverse transcriptase also served as controls for RT-PCR and produced no amplicon. After we determined that no contaminating genomic DNA existed in our cDNA, we performed RT-PCR. Relative quantities of expression using their respective primers (Fig. 8) were determined using an Mx3000P Real-Time PCR system following manufacturer's instructions (Stratagene; La Jolla, CA). DNA accumulation was measured using SYBR Green and ROX was used as reference dye. Only one product was present in each reaction as indicated by the SYBR Green dissociation curves of amplified products and by assay of terminal reactions by gel electrophoresis in 1% TBE agarose, thus ensuring that the product was of proper size. Template DNA was denatured for 10 minutes at 96°C, followed by PCR cycling temperatures set for denaturing for 30 seconds at 96°C, annealing for 60 seconds at 55°C and extension for 30 seconds at 72°C. The standard curve for the expression comparisons was constructed from the J2 stage sample. The J2 stage sample was diluted over a five-log range and used in parallel RT-PCR assays. All RT-PCR assays were conducted in triplicate. Threshold cycle (C_t_) values were plotted against the dilution series. PCR efficiencies were equal between the target and endogenous control. C_t _values and relative abundance were calculated using software supplied with the Mx3000P Real-Time PCR system. Our RT-PCR data was standardized against an est (CB380016) determined to experience no change in expression during *H. glycines *development. The relative abundance of mRNA was compared to that of CB380016 in the different sample types to calculate fold change. For each gene, a ratio was established between the control (CB380016) and the gene of interest (GOI) for the egg J2, 15 dpi and 30 dpi samples. To calculate fold expression, the ratio between CB380016 and the GOI at 15 dpi was set to a value of one. Other fold expression values for egg, J2 and 30 dpi were calculated using the ratio obtained at 15 dpi for GOI as the denominator. The ratio of the GOI for egg, J2 and 30 dpi, respectively, was used as the numerator. The value obtained after calculation was fold expression for those time-points. Standard error was used in the analyses.

## Abbreviations

est, expressed sequence tag; SCN, soybean cyst nematode; DMD, Duchenne muscular dystrophy; LIM, lin-11, isl-1, mec-3; CeHg, *C. elegans H. glycines *database; uncoordinated, *unc*; zinc finger, Zn^+2^-finger; RT-PCR, real-time quantitative PCR; nt, nucleotide; bp, base pair; J2, second stage juvenile; dpi, days post inoculation
